# Child growth faltering dynamics in food insecure districts in rural Ethiopia

**DOI:** 10.1111/mcn.13262

**Published:** 2021-09-15

**Authors:** Kalle Hirvonen, Abdulazize Wolle, Arnaud Laillou, Vincenzo Vinci, Stanley Chitekwe, Kaleab Baye

**Affiliations:** ^1^ Development Strategy and Governance Division International Food Policy Research Institute (IFPRI) Addis Ababa Ethiopia; ^2^ Economics Department State University of New York at Albany Albany New York USA; ^3^ Nutrition section UNICEF Ethiopia Addis Ababa Ethiopia; ^4^ Social Policy Section UNICEF Addis Ababa Ethiopia; ^5^ Center for Food Science and Nutrition. College of Natural Sciences Addis Ababa University Addis Ababa Ethiopia; ^6^ Research center for Inclusive Development in Africa (RIDA) Addis Ababa Ethiopia

**Keywords:** diet quality, growth faltering, infants, stunting, wasting

## Abstract

Child undernutrition disproportionally affects children in low‐ and middle‐income countries. In Ethiopia, both wasting and stunting are serious public health concerns, with high human and economic costs. Understanding the dynamics in ponderal and linear growth faltering is critical to inform the design of innovative interventions that can prevent both wasting and stunting in poor and complex settings. Using two longitudinal studies conducted in 2017 and 2019 in four highland regions of Ethiopia, we evaluated the dynamics and drivers of child growth faltering in children 6–23 months of age (*N* = 5003). Child wasting prevalence peaked during the first 6 months of life, whereas stunting increased significantly after 6 months of age. Male sex, child illnesses (i.e., diarrhoea or fever) and low consumption of fruits and vegetables were associated with higher odds of acute undernutrition (*P* < 0.05). The consumption of animal source foods (ASF) was associated with increases (β: 95% CI) in weight‐for‐length *Z*‐score (WLZ; 0.12: 0.0002; 0.242), whereas fruit or vegetables consumption was associated with increases in midupper arm circumference (MUAC; 0.11 cm: 0.003; 0.209). Only consumption of ASF was the statistically significant predictor of future linear growth (0.14: 0.029; 0.251). Distinct trends in WLZ and MUAC were observed by child sex and age. Improving diet quality through improved nutrition knowledge and increased access and affordability of ASFs, along with effective infection prevention/control measures could prevent both child wasting and stunting concurrently.

**Key messages**

- Ponderal and linear growth faltering have several shared drivers.
- Consumption of animal source foods in 6–23 months of age is associated with lower odds of subsequent linear growth faltering.
- Midupper arm circumference and weight‐for‐length *Z*‐score appear to identify children with different manifestations of acute malnutrition.

## 1 INTRODUCTION

Globally, 144 million children under 5 years of age were estimated to be stunted and 47 million wasted in 2020 (UNICEF, WHO, & World Bank, 2020). Child undernutrition disproportionally affects children in low‐ and middle‐income countries, with the regions of sub‐Saharan Africa and South‐Asia contributing the largest share of stunted and wasted children (UNICEF et al., 2020). Recognising the poor cognitive, health and productivity outcomes associated with child undernutrition (Alderman et al., 2006), the World Health Assembly nutrition targets for 2025 aimed for a 40% reduction in the number of stunted children and committed to reducing child wasting to a prevalence of <5% (WHO, 2014). This high level attention, further consolidated by the adoption of the targets by the 2030 sustainable development goals (SDGs), galvanised the set‐up and scale‐up of nutrition‐specific and nutrition‐sensitive interventions in many countries (Scott et al., 2020).

Interventions targeting child wasting and child stunting have traditionally been viewed as requiring two distinct approaches (Laillou et al., 2020; Wells et al., 2019). Child wasting considered as an acute form of malnutrition, its severe forms are treated in health centres or in the community using specialised therapeutic regimens (F75/100 milks and ready to use therapeutic foods), whereas interventions addressing stunting are preventive in nature and focus on addressing immediate and underlying causes of linear growth faltering (Laillou et al., 2020). However, more recent studies have argued that the two conditions (stunting and wasting) may be linked and are part of a continuum or a sequence of events (Wells et al., 2019). Indeed, a bidirectional relationship where child wasting contributes to subsequent stunting, as well as child stunting contributing to wasting relapse after recovery, has been observed (Stobaugh et al., 2018). However, more studies from contexts that have high burden of wasting and stunting are needed to confirm these findings.

In Ethiopia, both wasting and stunting remain serious public health concerns, with high human and economic costs (Baye & Hirvonen, 2020a). While stunting prevalence has declined substantially over the past two decades, 38% of children under 5 were stunted in 2016 (CSA & ICF, 2016). In 2016, 10% of the children in the same age range were categorised as wasted at the time when the Demographic Health Survey was administered (CSA & ICF, 2016). More than 100 million USD is being spent annually for treatment of acute malnutrition in the country, which combined with the enormous health, cognitive, and productivity costs associated with wasting and stunting is likely to severely constrain Ethiopia's ambition to prosper (Laillou et al., 2020). Besides, the wide gap in the prevalence of stunting between the poorest and the wealthiest segment of the population implies that inequalities are likely to widen if bold actions that close these gaps are not implemented (Baye, 2021; Baye et al., 2020).

We used two longitudinal surveys conducted in 2017 and 2019 to evaluate the dynamics of ponderal growth faltering and identified drivers of change to length‐for‐age *Z*‐score. The data used in the analysis come from the districts in which Ethiopia's Productive Safety Net Program (PSNP) operate. Over the years, the rates of wasting and stunting in these localities have showed little change and remain extremely high (Berhane et al., 2015, 2020), calling for a better understanding of the dynamics in ponderal and linear growth faltering to inform the design of innovative interventions that can prevent both wasting and stunting in poor and complex settings where the PSNP operates.

## 2 METHODS

### 2.1 Context

This study focused on the four highland regions of Ethiopia: Amhara, Oromia, Southern Nations, Nationalities and Peoples' Region (SNNP‐including the newly formed Sidama region) and Tigray, which represent more than 80% of the total population in the country (Central Statistical Agency [Ethiopia], 2010). Despite significant progress over the last decade, chronic and acute undernutrition rates in these regions remain high. In 2016, stunting prevalence ranged between 36.5% in Oromia and 46.3% in Amhara, while prevalence of child wasting ranged between 6.0% (in SNNP) and 11.1% in Tigray (CSA & ICF, 2016).

This study further focused on areas within these four regions in which the PSNP operates. With 8 million beneficiaries, PSNP is one of the largest safety net programmes in the world (World Bank, 2018). Geographically, the program operates in chronically food insecure districts (*woredas*). After the district selection, communities themselves select the beneficiaries targeting chronically food insecure households. Beneficiary households receive cash or food (mostly in the form of cereals) payments for undertaking public works. Households with limited labour capacity receive unconditional payments. In terms of magnitude, the PSNP transfers are estimated to be equivalent to about 15% of household consumption (Hirvonen & Hoddinott, 2021). While household food security has been improving in these localities, partly due to the PSNP (Berhane et al., 2016), this has not translated to better anthropometric outcomes for young children (Berhane et al., 2015, 2020). Chronic undernutrition prevalence among young children in the PSNP districts in the highland regions remains extremely high, and intra‐annual fluctuations in child weights relative to their heights are large (Baye & Hirvonen, 2020b; Hirvonen & Headey, 2018). Moreover, the differences in children's anthropometric and dietary outcomes between households benefitting from the PSNP and other poor households are negligible (Berhane et al., 2020).

### 2.2 Data collection and measures

This study was based on a secondary analysis of two longitudinal surveys administered in 2017 and 2019 in the four highland region districts in which the PSNP operates. The primary purpose of these surveys was to obtain pre‐intervention (2017) and post‐intervention (2019) information for an evaluation of nutrition sensitive components of the PSNP (Berhane et al., 2020). A stratified sample was drawn from areas in which the PSNP operates in the four highland regions. The household sample was restricted to poor households with a child less than 24 months of age in March 2017 or March 2019 (see supporting information S1 for more details about the sampling strategy). In 2017, 2635 children who were less than 24 months of age in March were measured twice within a 6‐month interval, first in March and then in August. In 2019, a new stratified random sample of 2626 children who were less than 24 months of age in March was drawn from the same enumeration areas and these children were measured twice, first in March and then in August.

### 2.3 Variables

Heights, weights and midupper arm circumference (MUAC) were measured from all children following the WHO and UNICEF guidelines (WHO & UNICEF, 2009). MUAC measures for children less than 6 months of age were not considered in the analysis. Children's lengths and weights were converted to *Z*‐scores using the WHO growth standards (de Onis et al., 2007; WHO, 2006). As primary measures of acute undernutrition, we used weight‐for‐length *Z*‐score (WLZ) and MUAC as well as a binary indicator classifying children as acutely undernourished if WLZ < −2 or MUAC < 12.5 cm (UNHCR & WFP, 2011). As a primary measure of chronic undernutrition, we used length‐for‐age *Z*‐score (LAZ). After removing biologically implausible measurements (those below −6.0 and above 6.0 for LAZ and those below −5.0 and above 5.0 for WLZ), we had 10,006 anthropometric (length, weight or midupper arm circumference; MUAC) records from 5003 children who were measured in both March and August survey rounds. Supporting information S2 provides more information about the anthropometric measurements.

As independent variables, we focused on immediate causes of child malnutrition (UNICEF, 1998): inadequate dietary intake and morbidities. As measures of inadequate dietary intake, we focused on infant and young child feeding (IYCF) practices. More specifically, as IYCF indicators, we used binary variables capturing children who consumed from specific food groups and child's meal frequency in the 24 h prior to the interview. Due to the extremely low dietary diversity in this context (see Table 1), we regrouped the seven IYCF food groups (WHO, 2008) into four food groups: staples, pulses, animal sourced foods (ASF) and fruits and vegetables. As a measure of child morbidity, we used a binary variable obtaining a value of 1 if the child was reported to have had fever or diarrhoea in the past 2 weeks preceding the interview.

**Table 1:** Child dietary indicators and prevalence of illnesses in March, children 6–23 months of age, *N* = 3771

Indicator	% /mean
**Child dietary indicators in the past 24 h:**	
Dietary diversity score, mean	1.5
Child consumed grains, roots or tubers, %	78.4
Child consumed legumes or nuts, %	27.7
Child consumed any animal source foods, %	13.0
*Child consumed dairy products*, %	*7*.*1*
*Child consumed poultry*, *fish or meat*, %	*1*.*6*
*Child consumed eggs*, %	*5*.*1*
Child consumed any fruit or any vegetables, %	27.8
*Child consumed vitamin‐A rich fruit or vegetables*, %	*5*.*9*
*Child consumed other fruit or vegetables*, %	*26*.*0*
Meal frequency, mean	2.0
**Child illnesses in the past 2 weeks:**	
Child had fever or diarrhoea, %	40.6
*Child had fever*, %	*32*.*0*
*Child had a diarrhoea*, %	*25*.*8*

*Note*: The italics was used to indicate that it is a subset.

As control variables, we used a host child, mother and household level variables capturing the underlying causes of child undernutrition (UNICEF, 1998). As child level variables, we used age (spline function with knots at 12 and 18 months) and sex. As maternal level variables, we used age, level of education, number of pregnancies and IYCF knowledge (see supporting information S3 for more details). Household level variables included characteristics of household head, household size, number of under 5 children in the household, household wealth, access to safe water and sanitation and year in which the household was interviewed. We also controlled for the region where the household was located. Supporting information S4 describes the variables used in the analysis.

### 2.4 Statistical analyses

The statistical analyses combined cross‐sectional and longitudinal data from 2017 and 2019.

To examine anthropometric trajectories with respect to child's age (Victora et al., 2010), we used locally weighted polynomial regressions that regressed child's anthropometric outcome (LAZ, WLZ or MUAC) on his/her age in months. We used Epanechnikov kernel‐density function weights in these regressions, and the bandwidth was selected using the rule‐of‐thumb method (Fan & Gijbels, 1995). These regressions were estimated using data collected in the March survey rounds with the sample restricted to children less than 24 months of age.

Multivariable ordinary least squares regression models were used to assess factors associated with child's WLZ, LAZ and MUAC. Logistic regression was used when the outcome variable was acute undernutrition. The sample in all multivariable regressions was restricted to children who were 6–23 months of age in March survey rounds.

Since WLZ and MUAC respond to nutritional inadequacies and infections in the period immediately preceding anthropometric measurement (Khara & Dolan, 2014), we used cross‐sectional data from the March survey rounds to assess the degree to which the immediate causes of child malnutrition were correlated with WLZ alone, MUAC alone and the binary indicator of acute undernutrition based on WLZ and MUAC. Meanwhile, LAZ captures cumulative growth deficits over a longer time period (Khara & Dolan, 2014). Therefore, we used change in child's LAZ between March and August as our outcome variable to analyse how the immediate causes of child malnutrition measured in March predicted linear growth over the subsequent 6‐month period. As a robustness check, we also estimated the same regression using change in raw (i.e., non‐standardised) lengths.

In all regressions, the independent variables at child, maternal and household levels were measured in March. We reported both unadjusted and adjusted coefficients. Standard errors were clustered at the district level. All statistical analyses were conducted using Stata, version 16.1 (StataCorp, Texas).

We also assessed if the associations of immediate causes of child malnutrition varied across different points of the WLZ or the MUAC distributions. While least squares regressions minimise variation around the mean, researchers working with anthropometric data often employ binary outcome variables such as WLZ < −2. Though intuitive in the terms of quantifying risk factors in terms of odds ratios, using dichotomous variables unnecessarily discards information leading to inefficiency and, therefore, is not recommended by medical statisticians (Royston et al., 2006). Considering this and the increased concern over the lack of biological basis for the cut‐offs used to dichotomise child anthropometric data (Leroy & Frongillo, 2019; Perumal et al., 2018), we used a quantile regression approach (Koenker & Hallock, 2001) that minimised absolute residual deviations around the 25th, 50th and 75th percentiles. These quantile regressions informed whether the correlation between poor IYCF practices or infections and WLZ (MUAC) varied depending on where the child is located in the conditional WLZ (MUAC) distribution. We further used interquantile range regressions to test whether the differences in the estimated coefficients at 25th and 75th percentiles were statistically different from zero. In our sample, 25th percentile value in the unconditional WLZ distribution in March was −1.33 SD and in the unconditional MUAC distribution 12.7 cm. The standard errors in all quantile and interquantile range regressions were computed via bootstrapping with 500 repetitions (Brownstone & Valletta, 2001).

## 3 RESULTS

Based on the pooled data from 2017 and 2019 March survey rounds, 12.4% of children 6–23 months were wasted (WLZ < −2 SD), 17.7% had MUAC less than 12.5 cm and 24.5% were acutely undernourished (WLZ < −2 SD or MUAC < 12.5 cm). Stunting (LAZ < −2) prevalence was 39.1%. Average child grew 4.2 cm between March and August rounds, which is 0.34 of a SD less than the median child of the same sex and age in the WHO (2006) growth standard, implying substantial growth faltering.

Average child 6–23 months consumed from 1.5 food groups in March. Nearly 80% of the children consumed starchy staples (grains, roots or tubers) and 28% legumes or nuts in the 24 h preceding the interview in March (Table 1). Consumption of animal source foods was not common; only 13% of the children consumed dairy products, flesh foods or eggs. Nearly 28% of the children consumed fruit or vegetables. Average meal frequency in the sample was two feedings. Dietary diversity and meal frequency increased with child age (supporting information S5). More than 40% of the children had either fever or diarrhoea in the past 14 days, with one third of the children reported to have experienced fever.

**Figure 1:**
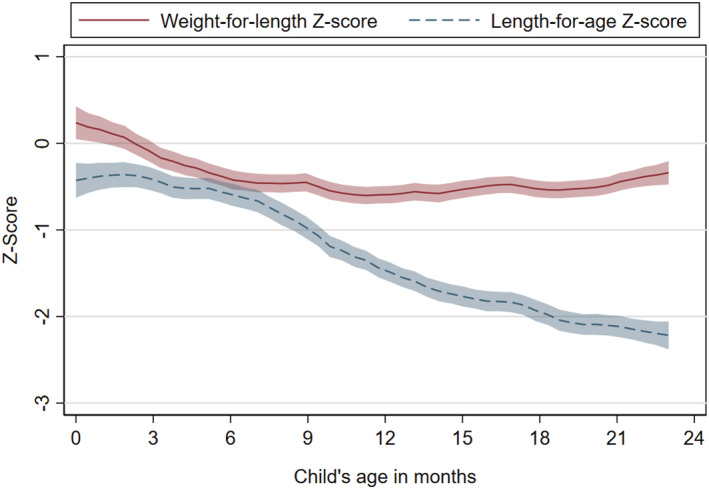
Relationship between child's age and weight‐for‐length *Z*‐score (WLZ)/length‐for‐age *Z*‐score (LAZ). *N* = 4876 children 0–23 months of age. March 2017 and 2019 rounds. Local polynomial regression. Shaded areas represent 95% confidence intervals

**Figure 2:**
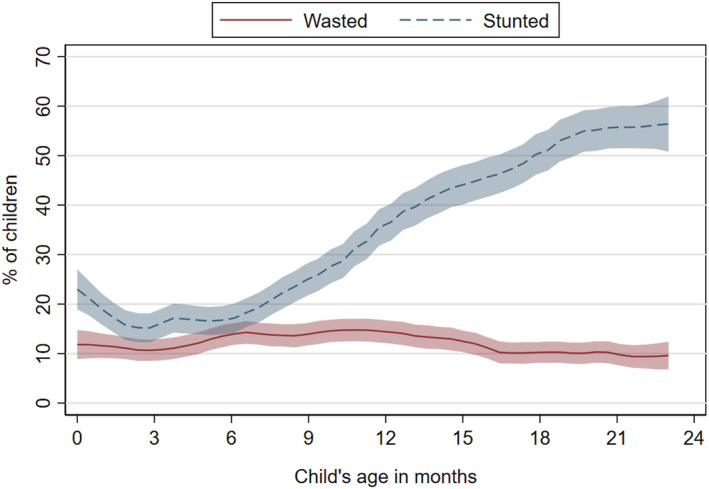
Relationship between child's age and child wasting/stunting. *N* = 4876 children 0–23 months of age. March 2017 and 2019 rounds. Local polynomial regression. Shaded areas represent 95% confidence intervals

Cross‐sectional data from March rounds indicated that WLZ decreased during 0–6 months of age after which it remained relatively stable while LAZ displayed a distinct age relationship, with sharp linear decrease between 6 and 23 months of age (Figure 1). MUAC increased monotonically with child age (supporting information S5). Wasting risk was slightly elevated for children 6–16 months of age (Figure 2). Child stunting prevalence was lowest at 2–6 months and then increased rapidly for older children (Figure 2). More than 50% of the children 18–23 months had LAZ < −2 (Figure 2).

Unadjusted and adjusted linear regressions showed that illnesses in the past 2 weeks were strongly correlated with both WLZ and MUAC (Table 2). Based on adjusted estimates, children who experienced fever or diarrhoea in the 2 weeks prior to the anthropometric measurements were taken had 0.21 SD units lower WLZ (95% CI: −0.291; −0.120) and had 0.13 cm smaller MUAC (95% CI: −0.230; −0.036), on average. The consumption of ASF was associated with a 0.12 increase in WLZ (95% CI: 0.0002; 0.242). Meanwhile, the consumption of fruit or vegetables was associated with 0.11 cm increase in MUAC (95% CI: 0.003; 0.209), on average. The association between other IYCF indicators (consumption of grains, roots or tubers; consumption of legumes or nuts; consumption of animal sourced foods; and child's meal frequency) and WLZ or MUAC was not statistically significant in adjusted models. Male children had lower WLZ and greater MUAC compared to female children. Mother's IYCF knowledge and household wealth (durable asset index) were positively associated with both WLZ and MUAC.

**Table 2:** Unadjusted and adjusted associations between immediate causes of child malnutrition and measures of acute nutritional status (WLZ and MUAC) in March

Outcome variable:	(1)	(2)	(3)	(4)
	Weight‐for‐length *Z*‐score		MUAC (in cm)	
Model type:	Unadjusted	Adjusted	Unadjusted	Adjusted
*N*:	3690	3681	3754	3743
Child had fever or diarrhoea	−0.227^***^ (0.044)	−0.208^***^ (0.044)	−0.147^**^ (0.050)	−0.131^**^ (0.049)
Child consumed grains, roots or tubers	0.119 (0.080)	0.098 (0.079)	0.112 (0.063)	0.083 (0.061)
Child consumed legumes or nuts	−0.004 (0.061)	0.001 (0.060)	0.064 (0.064)	0.068 (0.061)
Child consumed animal sourced foods	0.166^*^ (0.064)	0.121^*^ (0.061)	0.079 (0.056)	0.012 (0.053)
Child consumed fruit or vegetables	0.072 (0.066)	0.013 (0.059)	0.149^**^ (0.056)	0.108^*^ (0.051)
Child's meal frequency in the past 24 h	−0.015 (0.014)	−0.021 (0.013)	0.009 (0.014)	0.003 (0.013)
Male child		−0.145^***^ (0.042)		0.306^***^ (0.038)
Mother's age		−0.003 (0.006)		−0.009^*^ (0.005)
Mother has been to school		0.054 (0.062)		0.064 (0.048)
Number of pregnancies of mother		−0.031 (0.019)		−0.015 (0.017)
Mother's IYCF knowledge score		0.023^*^ (0.009)		0.030^**^ (0.010)
Male headed household		0.024 (0.080)		0.032 (0.064)
Household head has been to school		−0.096 (0.051)		−0.064 (0.051)
Household size		0.004 (0.021)		0.015 (0.017)
Number of under 5 years children in household		−0.118^***^ (0.032)		−0.059 (0.031)
Tropical livestock units owned		0.002 (0.014)		0.002 (0.013)
Durable asset index		0.048^**^ (0.018)		0.039^**^ (0.014)
Food gap over the last 6 months		−0.039 (0.022)		−0.001 (0.017)
Binary variable for survey year?	Yes	Yes	Yes	Yes
Binary variables for administrative regions?	Yes	Yes	Yes	Yes
Child's age in month spline variables?	Yes	Yes	Yes	Yes
*R* ^2^	0.026	0.048	0.054	0.086

*Note:* Standard errors clustered at the district level in parentheses. Sample restricted to children 6–23 months of age. All variables based on data collected in March 2017 or 2019 rounds.

Abbreviations: IYCF, infant and young child feeding; MUAC, midupper arm circumference; WLZ, weight‐for‐length *Z*‐score.

* Statistical significance denoted at *p* < 0.05.

** Statistical significance denoted at *p* < 0.01.

^***^ Statistical significance denoted at *p* < 0.001.

Illnesses in the past 2 weeks were also strongly positively associated with the odds of child being acutely undernourished (WLZ < −2 SD or MUAC < 12.5 cm) in March. Children who consumed fruit or vegetables were less likely to be acutely undernourished. Maternal nutritional knowledge and durable asset levels were negatively associated with the odds of child being acutely undernourished (supporting information S6).

The estimates based on quantile regression models showed that illnesses in the past weeks were strongly correlated with WLZ and MUAC across the full distribution and that these differences in the magnitude of these associations were not statistically significant (supporting information S7). The association between IYCF indicators and WLZ was not significant in the quantile regressions. Consumption of fruit and vegetables was positively associated with MUAC at 25th and 50th percentiles of the conditional MUAC distribution but not at the 75th percentile. However, the difference in coefficients between 25th and 75th percentiles was not statistically different from zero.

Illnesses experienced just before the March survey round were not associated with change in LAZ between March and August (Table 3). Out of the IYCF indicators, only consumption of ASF was the statistically significant predictor of future linear growth. Children who were reported to have consumed ASF in March round grew 0.14 LAZ units more between March and August than did other children (95% CI: 0.029; 0.251), on average and ceteris paribus. Mother's IYCF knowledge was also positively associated with linear growth during the subsequent 6 months (*p* < 0.001). Using raw, non‐standardised change in height instead of change in LAZ resulted in qualitatively similar findings (supporting information S8).

**Table 3:** Unadjusted and adjusted associations between immediate causes of child malnutrition and future growth faltering (change in LAZ between March and August)

Outcome variable:	(1)	(2)
	Change in length‐for‐age	
Model type:	Unadjusted	Adjusted
*N*:	3637	3681
Child had fever or diarrhoea	−0.041 (0.038)	−0.042 (0.038)
Child consumed grains, roots or tubers	0.048 (0.057)	0.042 (0.056)
Child consumed legumes or nuts	−0.038 (0.045)	−0.043 (0.045)
Child consumed animal sourced foods	0.167^**^ (0.055)	0.137^*^ (0.056)
Child consumed fruit or vegetables	−0.104 (0.055)	−0.094 (0.055)
Child's meal frequency in the past 24 h	−0.015 (0.012)	−0.016 (0.012)
Male child		0.053 (0.037)
Maternal age		−0.001 (0.005)
Has been to school		−0.023 (0.045)
Number of pregnancies		0.004 (0.017)
Mother's IYCF knowledge score		0.024^***^ (0.007)
Male headed household		−0.002 (0.053)
Household head has been to school		−0.063 (0.042)
Household size		−0.005 (0.015)
Number of under 5 years children in household		−0.076^*^ (0.033)
Tropical livestock units owned		0.016^*^ (0.006)
Durable asset index		−0.010 (0.010)
Food gap over the last 6 months		−0.010 (0.017)
Binary variable for survey year?	Yes	Yes
Binary variables for administrative regions?	Yes	Yes
Child's age in month spline variables?	Yes	Yes
*R* ^2^	0.088	0.096

*Note:* Standard errors clustered at the district level in parentheses. Sample restricted to children 6–23 months of age. All variables based on data collected in March 2017 or 2019 rounds, except the outcome variable that uses anthropometric data collected in March and August rounds in both years.

Abbreviations: IYCF, infant and young child feeding; LAZ, length‐for‐age *Z*‐score.

* Statistical significance denoted at *p* < 0.05.

** Statistical significance denoted at *p* < 0.01.

^***^ Statistical significance denoted at *p* < 0.001.

## 4 DISCUSSION

The present study highlighted that child wasting peaked during the first 6 months of life, whereas the prevalence of stunting increased significantly after 6 months of age. Male sex, child illnesses (i.e., diarrhoea or fever) and low consumption of fruits and vegetables were associated with higher odds of child being acutely undernourished (WLZ < −2 or MUAC < 12.5 cm) and lower MUAC, whereas consumption of ASFs and maternal IYCF knowledge was positively associated with WLZ and change in LAZ. The number of children under 5 years of age in a household was also negatively associated with both WLZ and LAZ, further suggesting that linear and ponderal growth faltering may have shared drivers. However, MUAC and WLZ appear to identify children with different manifestations of acute malnutrition.

The sharp decrease in WLZ score in the first 6 months, followed by a decrease in LAZ after 6 months, suggests that the sequence of events may be related. Indeed, recent studies have reported higher risk of growth faltering among infants and young children that have experienced wasting, and vice versa (Wells et al., 2019). In line with our observation, Mertens et al. (2020) showed that the peak incidence of wasting was in the first 3 months by pooling 18 cohort studies from LMICs. Illnesses like diarrhoea and fever, exacerbated by poor IYCF knowledge and practice, were associated with child wasting.

Recognising the potential synergies between ponderal and linear growth faltering, it is critical to identify drivers of both forms of undernutrition to design interventions that can address both wasting and stunting concurrently. Our analyses identified that low consumption of ASFs and maternal IYCF knowledge are key drivers that affect both children's ponderal and growth faltering. This is in line with previous studies that consistently found strong association between stunting and consumption of ASFs, but studies linking ASF to wasting are limited (Headey et al., 2018; Krasevec et al., 2017). The finding suggests that stunting prevention interventions could be designed in a way to contribute also to wasting prevention. For example, the promotion of dietary diversity, particularly the consumption of ASFs, could help prevent both wasting and stunting, provided that hygiene and food safety is ensured (Headey et al., 2018; Headey & Hirvonen, 2016; Hirvonen, Baye, et al., 2020). This could be achieved through food systems innovations that increase access and affordability of animal source foods (Abreha et al., 2021). In addition, improving maternal IYCF knowledge, which would require increasing the reach and quality of nutrition messaging through the health system (i.e., health extension workers), is critical (Abebe et al., 2016). However, the unaffordability of ASFs is likely to hinder their consumption among poor households in PSNP areas, indicating that efforts to increase the affordability of nutrient‐dense foods should be integral of wasting and stunting prevention approaches (Baye & Hirvonen, 2020a; Headey & Alderman, 2019; Hirvonen et al., 2017; Hirvonen, Bai, et al., 2020).

Noteworthy is also the difference in MUAC and WLZ trends by child age. While MUAC increased with age, the trend in WLZ was relatively stable. The discordance of WLZ and MUAC, with male children having lower WLZ but greater MUAC than female children, along with the difference in predictors of WLZ (ASF) and MUAC (fruits and vegetables), suggests that the two measures of acute malnutrition might be picking up on different forms/manifestations of acute malnutrition. Indeed, a recent study has confirmed this difference in MUAC and WLZ when used for admitting children into MAM treatment (Fabiansen et al., 2020). MUAC and WLZ performed differently for male and female children and also identified children with varying body composition (Fabiansen et al., 2020). This difference can have serious implications in the admission and cure rate of children (Guesdon & Roberfroid, 2019; Tessema et al., 2020).

A key strength of the study is the use of two longitudinal surveys conducted in different seasons to understand the dynamics of child wasting and stunting. The study has also allowed us to assess the degree to which same factors predict acute and chronic undernutrition. Nevertheless, our regression models could only explain a small fraction of the variation in WLZ, MUAC and change in LAZ. This was partly because our study population was a rather homogenous sample composed of the most vulnerable households. In addition, because of the observational design of our study, the findings suggest associations and should not be interpreted to imply causality.

Notwithstanding the above limitations, the present study highlighted that child morbidities are strongly associated with acute malnutrition (wasting) and that ASF consumption and maternal IYCF knowledge are associated with reduced risk of both ponderal and linear growth failure. Improving diet quality (including food safety) through improved IYCF knowledge, increased access and affordability of ASF and the implementation of infection prevention and control measures could help prevent both child wasting and stunting concurrently.

## CONFLICTS OF INTEREST

The authors declare that they have no conflicts of interest.

## AUTHOR CONTRIBUTIONS

KH and AW prepared and analysed the data. KH, KB and AL designed the research study with inputs from SC and VV. KH and KB wrote the paper with inputs from AL, SC and VV. All authors read and approved the final manuscript.

## Supporting information


**Table S2a** Percent of biologically implausible values, by anthropometric indicator and survey round
**Table S3a.** Correct responses to questions about IYCF practices in March rounds
**Table S4a.** Variable constructions
**Table S4b.** Summary statistics of all variables used in the analysis, N = 3,771
**Figure S5a.** Relationship between child's age and MUAC
**Figure S5b.** Relationship between child's age and acute undernutrition status
**Figure S5c.** Relationship between child's age and dietary diversity
**Figure S5d.** Relationship between child's age and meal frequency
**Table S6a.** Unadjusted and adjusted associations between immediate causes of child under‐nutrition and acute undernutrition (WLZ < −2 SD or MUAC < 12.5 cm) in March
**Table S7a.** Adjusted associations between immediate causes of child undernutrition and WLZ in March, quantile regression method
**Table S7b.** Adjusted associations between immediate causes of child undernutrition and MUAC in March, quantile regression method
**Table S8a.** Unadjusted and adjusted associations between immediate causes of child under‐nutrition and future growth faltering (change in non‐standardised child length between March and August)

## Data Availability

Data can be made available upon request.
