# Supplementary material for: Child growth faltering dynamics in food insecure districts in rural Ethiopia
Source: Matern Child Nutr. 2021 Sep 15;20(Suppl 5):e13262. doi: 10.1111/mcn.13262 (PMC11258764; doi:10.1111/mcn.13262)
Supplement: Supplementary file 1 — Table S2a Percent of biologically implausible values, by anthropometric indicator and survey round Table S3a. Correct responses to questions about IYCF practices in March rounds Table S4a. Variable constructions Table S4b. Summary statistics of all variables used in the analysis, N = 3,771 Figure S5a. Relationship between child's age and MUAC Figure S5b. Relationship between child's age and acute undernutrition status Figure S5c. Relationship between child's age and dietary diversity Figure S5d. Relationship between child's age and meal frequency Table S6a. Unadjusted and adjusted associations between immediate causes of child under‐nutrition and acute undernutrition (WLZ < −2 SD or MUAC < 12.5 cm) in March Table S7a. Adjusted associations between immediate causes of child undernutrition and WLZ in March, quantile regression method Table S7b. Adjusted associations between immediate causes of child undernutrition and MUAC in March, quantile regression method Table S8a. Unadjusted and adjusted associations between immediate causes of child under‐nutrition and future growth faltering (change in non‐standardised child length between March and August) [file MCN-20-e13262-s001.docx]

## Supplemental File S1: Sampling

The sampling for this survey was done in stages. First, 88 districts (woredas; 2nd lowest administrative level in Ethiopia) were selected randomly from the full list of PSNP districts in the four highland regions (Amhara, Oromia, SNNP and Tigray). Three sub-districts (kebeles; lowest administrative level) were randomly selected from each woreda. The Central Statistical Agency (CSA) of Ethiopia divides each sub-district into enumeration areas (EA) that are roughly of equal size, typically containing about approximately 200 households. From each sub-district, using the list of EAs provided to us by the CSA, we randomly selected one EA from each sub-district. The final step in the sampling procedure was to identify all households in the EA that had a child less than 24 months of age present in the household. If a young child was found residing in the household, the enumerators carefully probed his/her age to make sure that the child was indeed less than 24 months of age. Out of 46,866 households listed residing in the EAs, 10,318 households reported that they had a child less than 24 month of old.

Once the household listing was completed, we randomly selected 10 eligible households from each EA to be part of the survey. A household was eligible if it had a child less than 24-month-old. We further stratified the sample so that approximately half of the selected households were PSNP beneficiaries and half of them are poor (according to their self-assessment) but not benefitting from the program. Given the focus on poor households, this sample is not representative of the EAs or sub-districts in which the sample was drawn. In total, 2,635 households with children less than 24 months were visited in 88 woredas, 264 kebeles in Amhara, Oromia, SNNP and Tigray.

The original purpose of the survey was to serve as a baseline to the evaluation of the nutrition sensitive components of the PSNP. Reflecting this, the sample size and the number of clusters were based on statistical power calculations and assumptions about attrition that permit the evaluation team to detect a 10 percentage point increase in child’s consumption of dairy products, a 12 percentage point increase in the likelihood that a mother has at least four antenatal care visits and a 10 percent increase in women’s body-mass index.

## Supplemental File S2: Anthropometric measures

Anthropometric measures – height, weight and mid-upper arm circumference (MUAC) – were obtained for all index children. Enumerators were carefully trained by a qualified nurse to take these anthropometric measurements. The electronic questionnaire used during the fieldwork further alerted the enumerators for obvious outlier values and required them to re-measure the child if the height or weight value was biologically implausible. Moreover, given the focus on children 6-23 months of age, all heights (i.e. length) were measured lying down. Where possible, weights were obtained with children wearing light clothing.

Lengths, weights and MUACs were measured twice. In the analysis stage, biologically implausible measurements were first removed from the data and the average of the remaining measurements were used. The average lengths and weights were then converted to Z-scores using the WHO growth standards (de Onis et al., 2007; WHO, 2006). All anthropometric measures were calculated using the user-written *zanthro06* command (Leroy, 2011) in Stata 16.1.

Instructions on how to weigh children were widely followed, with more than 90 percent of children weighed wearing light or no clothing. Length of all children were measured lying down. Moreover, only few births are registered; in each round less than 15 percent of the children had a birth certificate and less than 20 percent had the child’s birth date recorded on a clinic card.^[[1]](#footnote-1)^ Therefore, in most cases, caregivers were aided by the enumerator to give their best recollection of when the child was born. Finally, we omitted children with biologically implausible Z-scores (those below -6.0 and above 6.0 for length for age Z-score and those below -5.0 and above 5.0 for weight-for-length and weight-for age z-scores) from the final data. Table S2a below shows the percent of children 6-23 months of age with biologically implausible values in each round.

Table S2a Percent of biologically implausible values, by anthropometric indicator and survey round

| **Survey round** | **LAZ (%)** | **WLZ (%)** | **WAZ (%)** |
| --- | --- | --- | --- |
| March 2017 | 1.4 | 3.0 | 1.0 |
| August 2017 | 0.9 | 1.2 | 1.2 |
| March 2019 | 0.7 | 1.2 | 0.9 |
| August 2019 | 1.0 | 1.2 | 0.9 |
| **All rounds** | **1.0** | **1.7** | **1.0** |

*Note: LAZ=Length-for-age Z score, WLZ=Weight-for-length Z score, WAZ=Weight-for-age Z score. Biologically implausible values were defined as LAZ: below -6.0 and above 6.0; WLZ: below -5.0 and above 5.0; and WAZ: below -5.0 and above 5.0.*

## Supplemental File S3: Description of the Construction of the Maternal Nutrition Knowledge Score

The nutrition knowledge module in the household questionnaire tested mothers' knowledge about complementary feeding through a battery of questions. The responses to the 14 questions were marked and the correct response to each question received one point. Table below displays the questions and the percent of mothers who answered correctly. We see that knowledge about breastfeeding related issues is relatively high but several knowledge gaps exist regarding complementary feeding related issues (e.g. sources of important nutrients, consequences of nutrient deficiencies). Overall, these findings resonate with earlier research on maternal nutrition knowledge in Ethiopia (Abebe, Haki, & Baye, 2016; Kim et al., 2015). We aggregated these responses into score. Each correct response was given one point, yielding an IYFC score ranging between 0 and 14.

Table S3a. Correct responses to questions about IYCF practices in March rounds

|  |  | **2017** | **2019** |
| --- | --- | --- | --- |
|  |  | **(%)** | **(%)** |
| 1 | Why exclusive breastfeeding is good for the baby * | 79 | 76 |
| 2 | What do with colostrum | 81 | 82 |
| 3 | Consequences of iron deficiency * | 52 | 46 |
| 4 | Sources of iron * | 35 | 36 |
| 5 | Consequences of vitamin A deficiency * | 38 | 39 |
| 6 | Sources of vitamin A * | 25 | 26 |
| 7 | What seasoning is often fortified with iodine | 57 | 52 |
| 8 | Are gruels traditionally prepared too thin | 14 | 18 |
| 9 | Food types to complement breastfeeding * | 70 | 58 |
| 10 | Can a 1-year old child eat alone without any supervision of an adult | 86 | 85 |
| 11 | Minimum meal frequency | 68 | 80 |
| 12 | What should a mother do when her child older than 6m has diarrhea * | 77 | 74 |
| 13 | How MUCH should a child be fed when s/he is sick | 28 | 31 |
| 14 | How OFTEN should a child be fed when s/he is sick | 31 | 34 |

* = more than one correct response; a point given if the respondent identified at least one of them.

## Supplemental File S4: Variable constructions and summary statistics

**Table S4a. Variable constructions**

| **Variable** | **Note on construction** |
| --- | --- |
| Weight-for-length Z-score | WLZ in March. |
| MUAC in cm | Mid-upper arm circumference (MUAC) in cm. |
| Change in length for age Z-score between March and August rounds | LAZ in August - LAZ in March. |
| Child had fever or diarrhea | Obtains a value 1 if child had a diarrhea or fever two weeks before the interview, zero otherwise. |
| Child consumed grains, roots or tubers | Obtains a value 1 if child consumed roots or tubers in the 24 hours before the interview, zero otherwise. |
| Child consumed legumes or nuts | Obtains a value 1 if child consumed legumes or nuts in the 24 hours before the interview, zero otherwise. |
| Child consumed any animal source foods | Obtains a value 1 if child consumed dairy, flesh foods or eggs in the 24 hours before the interview, zero otherwise. |
| Child consumed any fruit or any vegetable | Obtains a value 1 if child consumed fruit or vegetables in the 24 hours before the interview, zero otherwise. |
| Meal frequency | Number of times the child ate solid, semi-solid or soft foods in the 24 hours before the interview |
| **Child characteristics:** | **(All measured using data from the March round)** |
| Male child | Obtains value 1 if male child, zero otherwise. |
| Child age | Child's age in months. |
| **Maternal characteristics:** | **(All measured using data from the March round)** |
| Maternal age | Mother's age. |
| Has been to school | Obtains value 1 if mother has gone to school, zero otherwise. |
| Number of pregnancies | Number of pregnancies as reported by the mother. |
| IYCF knowledge score | Mother's responses to a nutrition knowledge test, min score = 0; max = 14. |
| **Household characteristics:** | **(All measured using data from the March round)** |
| Male head | Obtains value 1 if male head, zero otherwise. |
| Head has been to school | Obtains value 1 if head has gone to school, zero otherwise. |
| Household size | Number of household members. |
| Number of under 5 children in the household | Number of under 5 children residing in the household. |
| Tropical livestock units owned | Households' livestock holdings were converted to TLUs using conversion factors estimated in Jahnke (1982). |
| Durable asset index | Constructed using principal components methods |
| Food gap over the last 6 months | Household's subjective assessment of the number of months the household had difficulties in satisfying its food needs in the last 6 months |
| Located in the Amhara region | Obtains value 1 if the household is located in the Amhara region, zero otherwise. |
| Located in the Oromia region | Obtains value 1 if the household is located in the Oromia region, zero otherwise. |
| Located in the SNNP region | Obtains value 1 if the household is located in the SNNP region, zero otherwise. |
| Located in the Tigray region (reference) | Obtains value 1 if the household is located in the Tigray region, zero otherwise. Used as a reference category in the regressions. |
| Interviewed in 2017 | Obtains value 1 if the household was visited in 2017, zero otherwise. |
| Interviewed in 2019 | Obtains value 1 if the household was visited in 2019, zero otherwise. |

**Table S4b. Summary statistics of all variables used in the analysis, N = 3771**

| **Variable** | **mean** | **std. dev.** | **min** | **max** |
| --- | --- | --- | --- | --- |
| Weight-for-length/height Z-score | -0.50 | 1.38 | -5 | 4.93 |
| MUAC in cm | 13.5 | 1.21 | 8 | 19.8 |
| Change in length for age Z-score between March and August rounds | -0.34 | 1.16 | -3.93 | 3.94 |
| Child had fever or diarrhea | 0.41 | 0.49 | 0 | 1 |
| Child consumed grains, roots or tubers | 0.78 | 0.41 | 0 | 1 |
| Child consumed legumes or nuts | 0.28 | 0.45 | 0 | 1 |
| Child consumed any animal source foods | 0.13 | 0.34 | 0 | 1 |
| Child consumed any fruit or any vegetable | 0.28 | 0.45 | 0 | 1 |
| Meal frequency | 2.01 | 1.74 | 0 | 24 |
| **Child characteristics:** |  |  |  |  |
| Male child | 0.51 | 0.50 | 0 | 1 |
| Child age | 14.0 | 5.09 | 6 | 23 |
| **Maternal characteristics:** |  |  |  |  |
| Maternal age | 29.2 | 6.50 | 16 | 50 |
| Has been to school | 0.30 | 0.46 | 0 | 1 |
| Number of pregnancies | 3.82 | 2.29 | 1 | 15 |
| IYCF knowledge score | 7.44 | 2.91 | 0 | 14 |
| **Household characteristics:** |  |  |  |  |
| Male head | 0.89 | 0.31 | 0 | 1 |
| Head has been to school | 0.36 | 0.48 | 0 | 1 |
| Household size | 5.72 | 1.97 | 2 | 16 |
| Number of under 5 children in the household | 0.68 | 0.68 | 0 | 4 |
| Tropical livestock units owned | 3.23 | 3.78 | 0 | 65.4 |
| Durable asset index | 0.14 | 1.83 | -2.45 | 31.2 |
| Food gap over the last 6 months | 1.26 | 1.60 | 0 | 6 |
| Located in the Amhara region | 0.25 | 0.43 | 0 | 1 |
| Located in the Oromia region | 0.24 | 0.43 | 0 | 1 |
| Located in the SNNP region | 0.24 | 0.43 | 0 | 1 |
| Located in the Tigray region (reference) | 0.26 | 0.44 | 0 | 1 |
| Interviewed in 2017 | 0.51 | 0.50 | 0 | 1 |
| Interviewed in 2019 | 0.49 | 0.50 | 0 | 1 |

Note: std. dev. = standard deviation.

## Supplemental File S5: Additional local polynomial graphs

Figure S5a. Relationship between child's age and MUAC


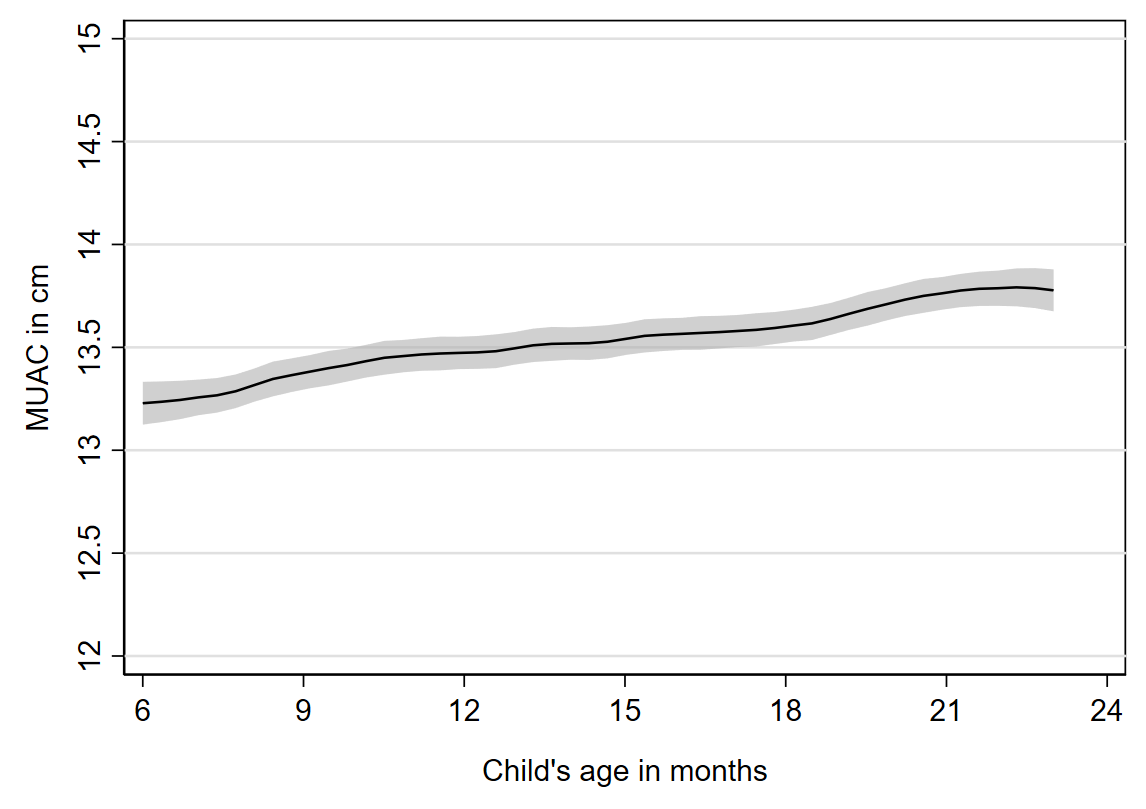


N = 3,754 children 6-23 months of age. March 2017 and 2019 rounds. Local polynomial regression. Shaded areas represent 95% confidence intervals.

Figure S5b. Relationship between child's age and acute undernutrition status


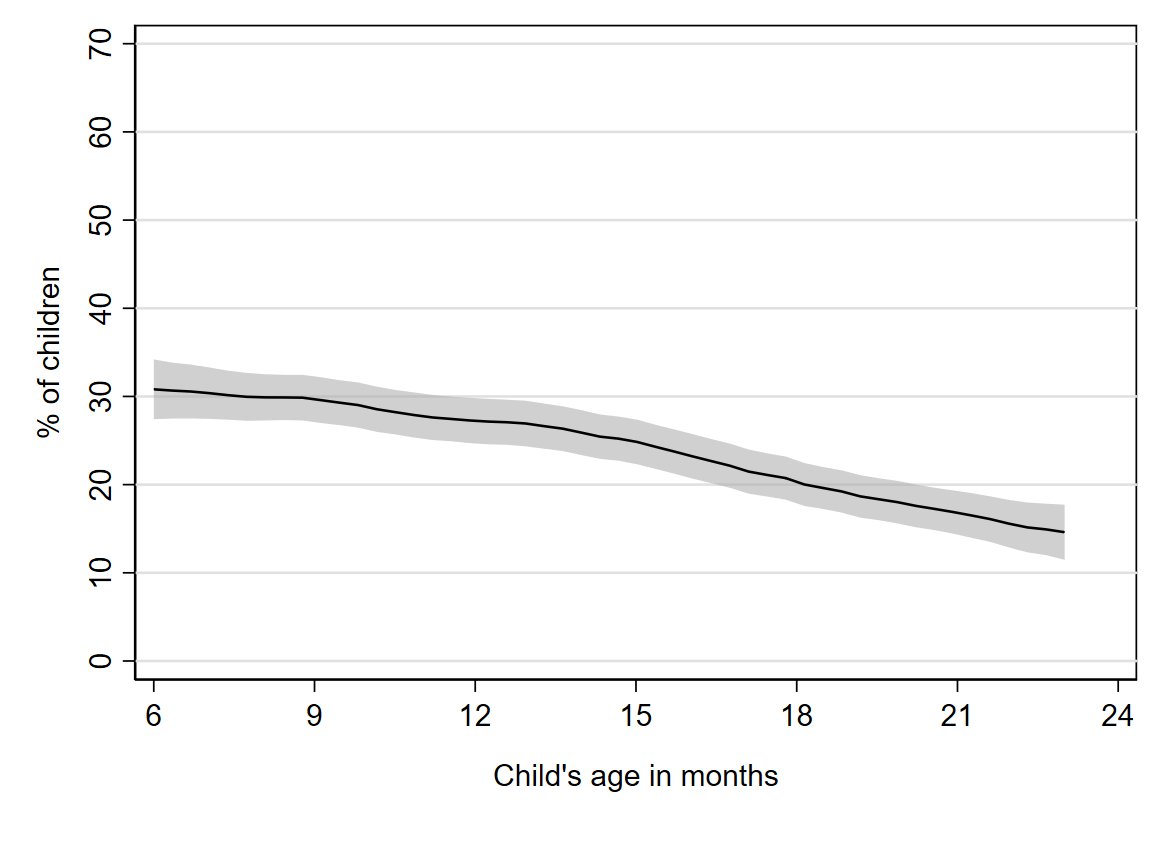


N = 3,765 children 6-23 months of age. March 2017 and 2019 rounds. Local polynomial regression. Shaded areas represent 95% confidence intervals. A child is classified as acutely undernourished if WLZ < -2 SD or MUAC < 12.5 cm.

**Figure S5c. Relationship between child’s age and dietary diversity**


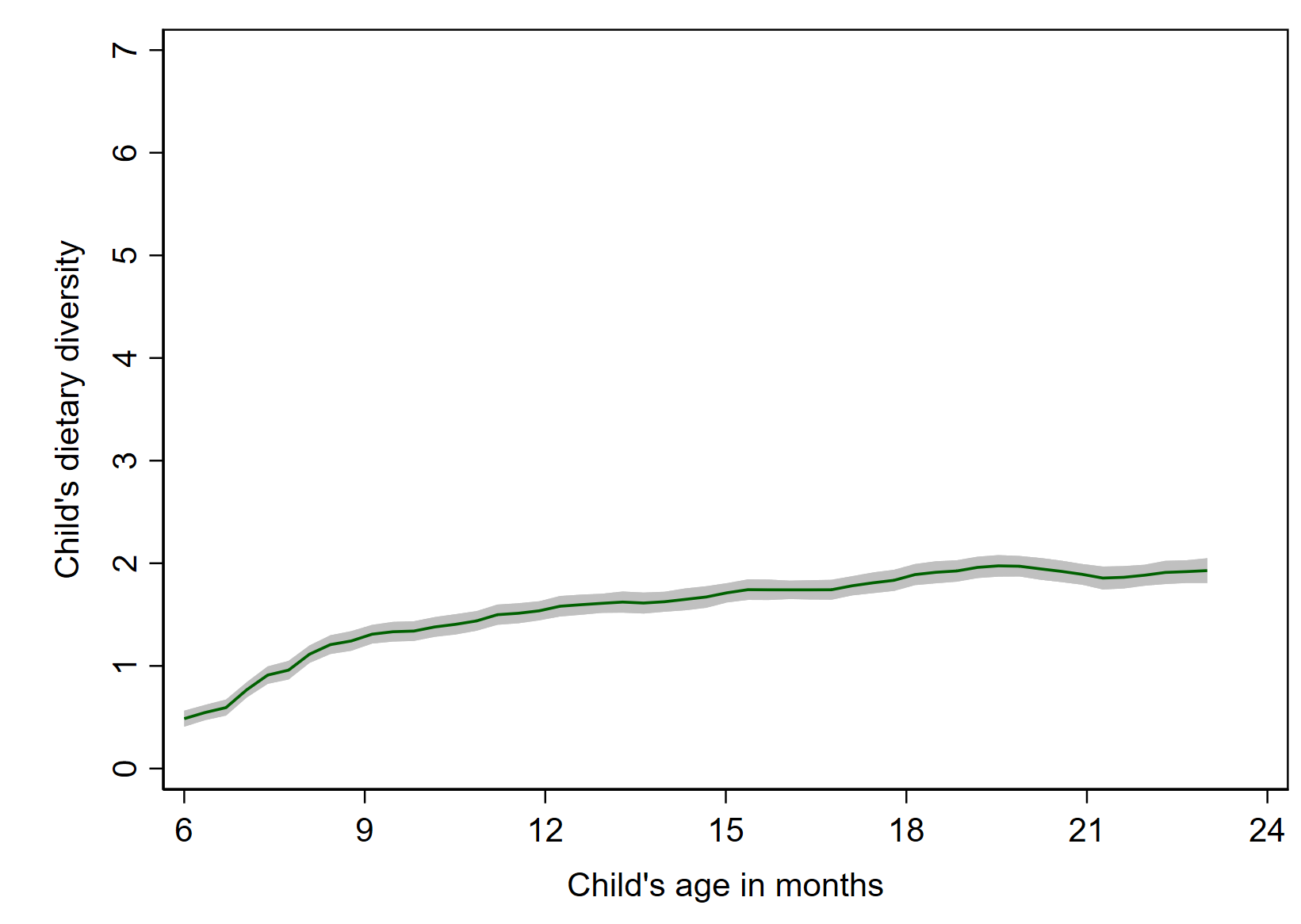


N = 3,775 children 6-23 months of age. March 2017 and 2019 rounds. Local polynomial regression. Shaded areas represent 95% confidence intervals.

**Figure S5d. Relationship between child’s age and meal frequency**


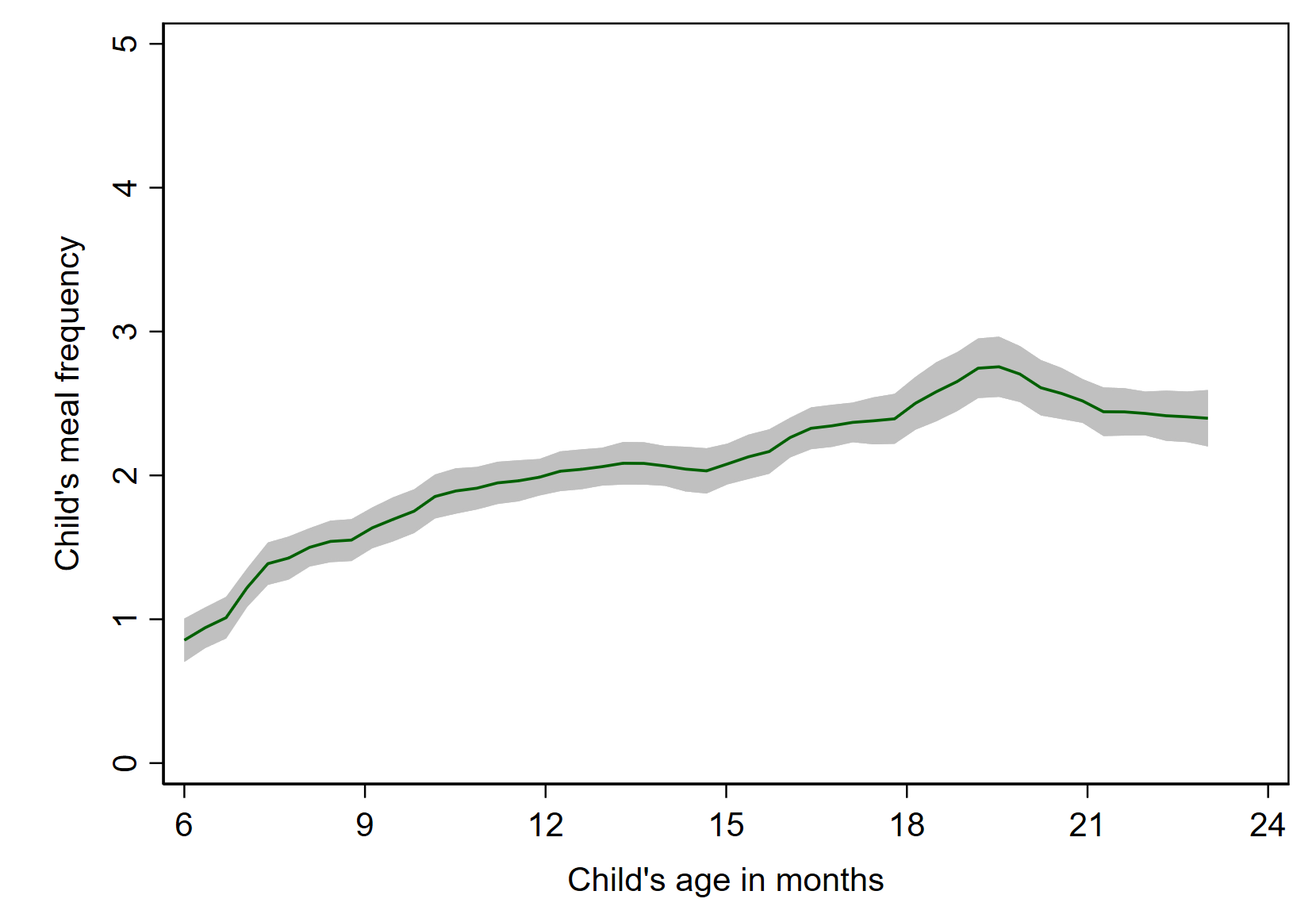


N = 3,775 children 6-23 months of age. March 2017 and 2019 rounds. Local polynomial regression. Shaded areas represent 95% confidence intervals.

## Supplemental File S6: Odds ratios for child being acutely undernourished

Table S6a. Unadjusted and adjusted associations between immediate causes of child under-nutrition and acute undernutrition (WLZ < -2 SD or MUAC < 12.5 cm) in March

|  | **(1)** | **(2)** |
| --- | --- | --- |
| N: | 3,765 | 3,754 |
| Child had fever or diarrhea | 1.350*** | 1.308** |
|  | (0.120) | (0.118) |
| Child consumed grains, roots or tubers | 0.937 | 0.962 |
|  | (0.103) | (0.103) |
| Child consumed legumes or nuts | 0.932 | 0.919 |
|  | (0.085) | (0.085) |
| Child consumed animal sourced foods | 0.839 | 0.889 |
|  | (0.102) | (0.108) |
| Child consumed fruit or vegetables | 0.753** | 0.818* |
|  | (0.075) | (0.079) |
| Child's meal frequency in the past 24 h | 0.994 | 1.002 |
|  | (0.030) | (0.029) |
| Male child |  | 0.891 |
|  |  | (0.073) |
| Mother's age |  | 1.003 |
|  |  | (0.009) |
| Mother has been to school |  | 0.908 |
|  |  | (0.087) |
| Number of pregnancies of mother |  | 1.046 |
|  |  | (0.035) |
| Mother's IYCF knowledge score |  | 0.969* |
|  |  | (0.015) |
| Male headed household |  | 0.869 |
|  |  | (0.112) |
| Household head has been to school |  | 1.055 |
|  |  | (0.122) |
| Household size |  | 0.952 |
|  |  | (0.031) |
| Number of under 5 children in household |  | 1.086 |
|  |  | (0.060) |
| Tropical livestock units owned |  | 1.010 |
|  |  | (0.013) |
| Durable asset index |  | 0.938* |
|  |  | (0.029) |
| Food gap over the last 6 months |  | 1.025 |
|  |  | (0.029) |
| Binary variable for survey year? | Yes | Yes |
| Binary variables for administrative regions? | Yes | Yes |
| Child's age in month spline variables? | Yes | Yes |

Note: Estimates are odds ratios from a logistic regression. Standard errors clustered at the district level in parentheses. Statistical significance denoted at ^*^ *p* < 0.05, ^**^ *p* < 0.01, ^***^ *p* < 0.001. Sample restricted to children 6-23 months of age. All variables based on data collected in March 2017 or 2019 rounds.

## Supplemental File S7: Quantile regression results

**Table S7a. Adjusted associations between immediate causes of child undernutrition and WLZ in March, quantile regression method**

|  | **(1)** | **(2)** | **(3)** | **(4)** |
| --- | --- | --- | --- | --- |
| **percentile:** | **25^th^** | **50^th^** | **75^th^** | **25^th^ vs 75^th^** |
| N: | 3,681 | 3,681 | 3,681 | 3,681 |
| Child had fever or diarrhea | -0.220*** | -0.272*** | -0.225*** | 0.042 |
|  | (0.061) | (0.060) | (0.054) | (0.063) |
| Child consumed grains, roots or tubers | 0.018 | 0.028 | 0.026 | 0.002 |
|  | (0.079) | (0.082) | (0.079) | (0.100) |
| Child consumed legumes or nuts | -0.006 | -0.042 | -0.053 | -0.027 |
|  | (0.065) | (0.061) | (0.060) | (0.087) |
| Child consumed animal sourced foods | 0.133 | 0.133 | 0.139 | -0.031 |
|  | (0.086) | (0.082) | (0.088) | (0.093) |
| Child consumed fruit or vegetables | 0.039 | 0.090 | 0.079 | 0.008 |
|  | (0.072) | (0.064) | (0.062) | (0.085) |
| Child's meal frequency in the past 24 h | -0.009 | -0.011 | -0.011 | 0.003 |
|  | (0.019) | (0.016) | (0.016) | (0.021) |
| Male child | -0.220*** | -0.187*** | -0.152** | 0.149* |
|  | (0.059) | (0.050) | (0.052) | (0.070) |
| Mother's age | -0.005 | -0.000 | -0.000 | 0.005 |
|  | (0.007) | (0.006) | (0.006) | (0.009) |
| Mother has been to school | 0.063 | 0.074 | 0.085 | -0.004 |
|  | (0.072) | (0.066) | (0.067) | (0.089) |
| Number of pregnancies of mother | -0.060** | -0.032 | -0.024 | 0.050 |
|  | (0.022) | (0.020) | (0.019) | (0.028) |
| Mother's IYCF knowledge score | 0.026* | 0.023* | 0.022* | -0.008 |
|  | (0.011) | (0.009) | (0.009) | (0.012) |
| Male headed household | 0.068 | 0.094 | 0.143 | -0.031 |
|  | (0.082) | (0.084) | (0.085) | (0.104) |
| Household head has been to school | -0.092 | -0.144* | -0.157** | 0.043 |
|  | (0.071) | (0.058) | (0.061) | (0.090) |
| Household size | 0.056** | -0.000 | -0.011 | -0.093*** |
|  | (0.018) | (0.018) | (0.017) | (0.025) |
| Number of under 5 children in household | -0.102* | -0.146*** | -0.126** | -0.029 |
|  | (0.051) | (0.044) | (0.043) | (0.056) |
| Tropical livestock units owned | -0.012 | -0.003 | -0.001 | 0.018* |
|  | (0.009) | (0.010) | (0.011) | (0.009) |
| Durable asset index | 0.053*** | 0.067*** | 0.061*** | -0.016 |
|  | (0.016) | (0.017) | (0.017) | (0.020) |
| Food gap over the last 6 months | -0.045* | -0.023 | -0.022 | 0.027 |
|  | (0.021) | (0.020) | (0.020) | (0.023) |
| Binary variable for survey year? | Yes | Yes | Yes | Yes |
| Binary variables for administrative regions? | Yes | Yes | Yes | Yes |
| Child's age in month spline variables? | Yes | Yes | Yes | Yes |

Note: Standard errors reported in parentheses and computed via bootstrapping (500 repetitions). Statistical significance denoted at * p < 0.05, ** p < 0.01, *** p < 0.001. Sample restricted to children 6-23 months of age. All variables based on data collected in March 2017 or 2019 rounds.

**Table S7b. Adjusted associations between immediate causes of child undernutrition and** **MUAC in March, quantile regression method**

|  | **(1)** | **(2)** | **(3)** | **(4)** |
| --- | --- | --- | --- | --- |
| **percentile:** | **25^th^** | **50^th^** | **75^th^** | **25^th^ vs 75^th^** |
| N: | 3,743 | 3,743 | 3,743 | 3,743 |
| Child had fever or diarrhea | -0.166** | -0.135** | -0.119* | 0.098 |
|  | (0.058) | (0.046) | (0.050) | (0.068) |
| Child consumed grains, roots or tubers | -0.012 | 0.076 | 0.107 | 0.091 |
|  | (0.068) | (0.063) | (0.068) | (0.093) |
| Child consumed legumes or nuts | -0.013 | 0.027 | 0.039 | 0.080 |
|  | (0.063) | (0.055) | (0.058) | (0.076) |
| Child consumed animal sourced foods | -0.044 | 0.007 | 0.030 | 0.123 |
|  | (0.083) | (0.073) | (0.077) | (0.088) |
| Child consumed fruit or vegetables | 0.165** | 0.141** | 0.111 | -0.075 |
|  | (0.063) | (0.054) | (0.058) | (0.075) |
| Child's meal frequency in the past 24 h | 0.021 | 0.014 | 0.007 | -0.013 |
|  | (0.019) | (0.014) | (0.015) | (0.021) |
| Male child | 0.294*** | 0.297*** | 0.306*** | -0.002 |
|  | (0.052) | (0.044) | (0.048) | (0.064) |
| Mother's age | -0.007 | -0.009 | -0.008 | -0.006 |
|  | (0.005) | (0.005) | (0.005) | (0.007) |
| Mother has been to school | 0.040 | 0.128* | 0.087 | 0.078 |
|  | (0.069) | (0.064) | (0.063) | (0.090) |
| Number of pregnancies of mother | -0.034 | -0.012 | -0.018 | 0.046 |
|  | (0.022) | (0.018) | (0.017) | (0.030) |
| Mother's IYCF knowledge score | 0.024** | 0.017* | 0.017 | 0.002 |
|  | (0.008) | (0.008) | (0.009) | (0.011) |
| Male headed household | 0.056 | 0.054 | 0.067 | -0.039 |
|  | (0.085) | (0.073) | (0.089) | (0.107) |
| Household head has been to school | -0.028 | -0.068 | -0.042 | -0.094 |
|  | (0.064) | (0.056) | (0.058) | (0.077) |
| Household size | 0.051* | 0.014 | 0.003 | -0.068* |
|  | (0.022) | (0.016) | (0.016) | (0.027) |
| Number of under 5 children in household | -0.089 | -0.051 | -0.009 | 0.076 |
|  | (0.045) | (0.040) | (0.039) | (0.052) |
| Tropical livestock units owned | -0.002 | 0.001 | 0.001 | 0.002 |
|  | (0.006) | (0.007) | (0.007) | (0.010) |
| Durable asset index | 0.043** | 0.058*** | 0.059*** | -0.006 |
|  | (0.016) | (0.015) | (0.015) | (0.018) |
| Food gap over the last 6 months | 0.002 | 0.003 | 0.004 | -0.011 |
|  | (0.018) | (0.015) | (0.015) | (0.021) |
| Binary variable for survey year? | Yes | Yes | Yes | Yes |
| Binary variables for administrative regions? | Yes | Yes | Yes | Yes |
| Child's age in month spline variables? | Yes | Yes | Yes | Yes |

Note: Standard errors reported in parentheses and computed via bootstrapping (500 repetitions). Statistical significance denoted at * p < 0.05, ** p < 0.01, *** p < 0.001. Sample restricted to children 6-23 months of age. All variables based on data collected in March 2017 or 2019 rounds.

## Supplemental File S8: Replicating Table 3 but using change in (non-standardized) child length as the outcome variable

Table S8a. Unadjusted and adjusted associations between immediate causes of child under-nutrition and future growth faltering (change in non-standardized child length between March and August)

|  | **(1)** | **(2)** |
| --- | --- | --- |
| **Outcome variable:** | **Change in child length (cm)** | |
| **Model type:** | **Unadjusted** | **Adjusted** |
| N: | 3,637 | 3,681 |
| Child had fever or diarrhea | -0.130 | -0.131 |
|  | (0.120) | (0.119) |
| Child consumed grains, roots or tubers | 0.164 | 0.155 |
|  | (0.181) | (0.175) |
| Child consumed legumes or nuts | -0.199 | -0.207 |
|  | (0.139) | (0.140) |
| Child consumed animal sourced foods | 0.544** | 0.473** |
|  | (0.164) | (0.166) |
| Child consumed fruit or vegetables | -0.216 | -0.197 |
|  | (0.153) | (0.155) |
| Child's meal frequency in the past 24 h | -0.033 | -0.035 |
|  | (0.035) | (0.035) |
| Male child |  | 0.046 |
|  |  | (0.105) |
| Maternal age |  | 0.005 |
|  |  | (0.014) |
| Has been to school |  | -0.032 |
|  |  | (0.140) |
| Number of pregnancies |  | -0.012 |
|  |  | (0.052) |
| Mother's IYCF knowledge score |  | 0.062** |
|  |  | (0.021) |
| Male headed household |  | 0.036 |
|  |  | (0.160) |
| Household head has been to school |  | -0.036 |
|  |  | (0.122) |
| Household size |  | 0.022 |
|  |  | (0.045) |
| Number of under 5 children in household |  | -0.208* |
|  |  | (0.091) |
| Tropical livestock units owned |  | 0.042** |
|  |  | (0.016) |
| Durable asset index |  | -0.032 |
|  |  | (0.031) |
| Food gap over the last 6 months |  | -0.019 |
|  |  | (0.050) |
| Binary variable for survey year? | Yes | Yes |
| Binary variables for administrative regions? | Yes | Yes |
| Child's age in month spline variables? | Yes | Yes |
| *R*^2^ | 0.023 | 0.030 |

Note: Standard errors clustered at the district level in parentheses. Statistical significance denoted at ^*^ *p* < 0.05, ^**^ *p* < 0.01, ^***^ *p* < 0.001. Sample restricted to children 6-23 months of age. All variables based on data collected in March 2017 or 2019 rounds, except the outcome variable that uses anthropometric data collected in March and August rounds in both years.

## Supplement References

Abebe, Z., Haki, G. D., & Baye, K. (2016). Health Extension Workers’ Knowledge and Knowledge-Sharing Effectiveness of Optimal Infant and Young Child Feeding Are Associated With Mothers’ Knowledge and Child Stunting in Rural Ethiopia. *Food and Nutrition Bulletin, 37*(3), 353 - 363.

de Onis, M., Onyango, A. W., Borghi, E., Siyam, A., Nishida, C., & Siekmann, J. (2007). Development of a WHO growth reference for school-aged children and adolescents. *Bulletin of the World Health Organization, 85*(9), 660-667.

Jahnke, H. E. (1982). *Livestock production systems and livestock development in tropical Africa* (Vol. 35): Kieler Wissenschaftsverlag Vauk Kiel.

Kim, S. S., Ali, D., Kennedy, A., Tesfaye, R., Tadesse, A. W., Abrha, T. H., . . . Menon, P. (2015). Assessing implementation fidelity of a community-based infant and young child feeding intervention in Ethiopia identifies delivery challenges that limit reach to communities: a mixed-method process evaluation study. *BMC Public Health, 15*(1), 316.

Leroy, J. (2011). ZSCORE06: Stata module to calculate anthropometric z-scores using the 2006 WHO child growth standards. *Statistical Software Components*.

WHO. (2006). WHO Child Growth Standards based on length/height, weight and age. *Acta Paediatrica, Suppl 450*, 76-85.

1. In March 2017, 14 % of the children had a birth certificate and 7.6 % had their date of birth recorded on a clinic card. The corresponding percentages in 2019 were 6.4 % with birth certificate and 18.8 % with a date of birth on a clinic card. [↑](#footnote-ref-1)
